# Statin Use and Influenza Vaccine Effectiveness in Persons >65 Years of Age, Taiwan

**DOI:** 10.3201/eid2606.190646

**Published:** 2020-06

**Authors:** Lung-Wen Tsai, Yung-Tai Chen, Chia-Jen Shih, Shuo-Ming Ou, Pei-Wen Chao, Shih-Hsiu Lo

**Affiliations:** Taipei Medical University Hospital, Taipei, Taiwan (L.-W. Tsai, P.-W. Chao, S.-H. Lo); Taipei City Hospital, Taipei (Y.-T. Chen);; University of Taipei, Taipei (Y.-T, Chen);; National Yang-Ming University, Taipei (Y.-T. Chen, C.-J. Shih, S.-M. Ou);; Taipei Veterans General Hospital, Taipei (S.-M. Ou)

**Keywords:** critical illness, vaccine-preventable diseases, influenza, hospitalization, vaccines, viruses, respiratory diseases, Taiwan

## Abstract

Debates on whether statin use reduces the effectiveness of influenza vaccines against critical illness and death among persons >65 years of age continue. We conducted a study of 9,427,392 persons >65 years of age who did and did not receive influenza vaccinations during 12 consecutive influenza seasons, 2000–01 through 2011–12. Using data from Taiwan’s National Health Insurance Research Database, we performed propensity score-matching to compare vaccinated persons with unvaccinated controls. After propensity score-matching, the vaccinated group had lower risks for in-hospital death from influenza and pneumonia and for hospitalization for pneumonia and influenza, circulatory conditions, and critical illnesses compared with the unvaccinated group. We stratified the 2 groups by statin use and analyzed data by interaction analysis and saw no statistically significant difference. We found that influenza vaccine effectively reduced risks for hospitalization and death in persons >65 years of age, regardless of statin use.

## Abstract 2

Influenza vaccine effectively reduced risks for in-hospital death or hospitalization, regardless of statin use.

Epidemics of influenza occur nearly every winter and last through spring, causing an average of 226,054 influenza-related hospital admissions and 51,203 influenza-related deaths in the United States annually (*1*–*3*). Persons >65 years of age are at greater risk for serious complications of influenza and ≈90% of deaths due to influenza and pneumonia occur among this age group (*1*,*4*). Taiwan, like other high-income countries, recognizes the importance of influenza vaccination and strongly recommends annual vaccination to prevent complications of influenza and reduce hospitalization rates and death in older persons (*5*,*6*).

Persons >65 years of age also are at greater risk for coronary atherosclerosis and cardiovascular disease. Statin treatment in this population is crucial, but benefits and risks should guide its use (*7*,*8*). In addition to cholesterol-lowering effects that provide cardiovascular benefits, statins have been shown to suppress T-cell activation and exhibit antiinflammatory and immunomodulatory properties (*9*–*12*). Few studies have investigated the effect of statins on vaccine effectiveness, but concerns have been raised that statins might interfere with the immune response to influenza vaccines and seem to reduce their effectiveness (*13*,*14*). A study of 6,961 trial participants >65 years of age from Colombia, Panama, the Philippines, and the United States showed that hemagglutination-inhibiting geometric mean titers to influenza strains were much lower in chronic statin users compared with nonusers (*13*). Another large-scale retrospective cohort study based on a research database covering influenza seasons for 2002–2011 in the United States revealed reduced influenza vaccine effectiveness against respiratory illness in statin users (*14*). By contrast, data from another retrospective 5-year cohort study of 1,403,651 statin users matched to nonusers found that use of statins around the time of influenza vaccination does not dramatically affect the risk for influenza-related visits and influenza-related hospitalizations in older adults (*15*). Another large-scale nationwide population study evaluated whether statin therapy reduced vaccination effectiveness in terms of influenza-associated critical illness hospitalizations and death and suggested high-dose influenza vaccines or vaccines containing adjuvants to boost the immune response might be needed in older populations (*16*). However, previous studies did not match cases and controls for characteristics, underlying health conditions, or concomitant drug use and did not focus on the outcomes of influenza-related critical illness and death.

We designed a large-scale, nationwide, population-based cohort study to explore heterogeneity of influenza vaccine effectiveness between statin users and nonusers among persons ≥65 years of age in Taiwan. We assessed risks for hospitalization for pneumonia and influenza, circulatory conditions, or critical illness and for in-hospital death and in-hospital death from pneumonia in this age group. We compared the vaccinated group with propensity score-matched control subjects who did not receive influenza vaccinations.

## Methods

### Data Source

We used the data from Taiwan’s National Health Insurance Research Database (NHIRD), which has been described in detail elsewhere (*17*–*19*). We extracted medical data for persons >65 years of age in Taiwan from an NHIRD dataset based on a regulation that prohibits use of the maximal amount of claims data and permits use of data from only one third of older beneficiaries for research purposes. Our dataset included information on all inpatient, emergency department, and outpatient visits; diagnosed illnesses and conditions; prescriptions; and procedures for one third of all persons >65 years of age in Taiwan. We used diagnostic and procedural codes from the International Classification of Diseases, 9th Revision, Clinical Modification (ICD-9-CM; https://www.cdc.gov/nchs/icd/icd9cm.htm) to ascertain details associated with inpatient and outpatient encounters. Because patient information in the NHIRD is secondary, deidentified, and encrypted, this study was exempted from a full ethics review by the institutional review board of Taipei Medical University Hospital (IRB no. 105TMUH-SP-07).

### Study Population

The study period encompassed 12 consecutive influenza seasons from 2000–01 through 2011–12 (*14*). The study sample was comprised of persons >65 years of age who resided in Taiwan during 2000–2012. Persons >65 years of age in Taiwan are encouraged to receive influenza vaccines, which are covered by insurance, between October 1 and December 31 each year. For our study, we defined the index date as the date of influenza vaccination for the vaccinated group. To avoid immortal time bias, for the unvaccinated group we randomly assigned index dates that corresponded to those in the vaccinated group. Because the same persons could be part of the unvaccinated group initially and later change to the vaccinated group during the influenza period each year, we did not include the unvaccinated period in our outcome analyses. In each year, we traced participants’ NHIRD records from January 1 through September 30 or death. 

### Statin Exposure

For each year in the study period, we identified all drug prescriptions written for participants before the index date by using inpatient and ambulatory care order files. For statin users, we identified persons who had initial dispensing date of statin on or before the index date. We defined chronic statin users as those who received and filled a prescription for a statin medication for >30 days (*20*,*21*).

### Outcomes

The outcomes of interest were in-hospital death and in-hospital death from pneumonia. Our analyses also included severe complications of influenza infections, including hospitalization for pneumonia, circulatory condition, and critical illness. We defined critical illness as hospitalization for acute respiratory failure (ICD-9-CM codes 518.5, 518.81, 518.82, or 96.7) or severe sepsis (ICD-9-CM codes 995.92 or 785.52) or organ dysfunction (*22*).

### Statistical Analyses

We examined the differences of baseline characteristics between the vaccinated group and unvaccinated group by using standardized mean differences. For each participant, we calculated a propensity score for the likelihood of receiving influenza vaccination by using baseline covariates in a multivariate logistic regression model (Appendix Table 1). For each person in the vaccinated group, we identified 1 person in the unvaccinated group that was frequency-matched according to propensity score (*23*). We used Cox regression with adjusted imbalance covariates to calculate hazard ratios (HRs) for in-hospital death; in-hospital death from pneumonia; and hospitalization for pneumonia, circulatory conditions, and critical illness. We conducted a subgroup analyses and used a likelihood ratio test to explore heterogeneity of vaccine effectiveness between statin users and nonusers. We used SQL Server 2012 (Microsoft, https://www.microsoft.com) for data linkage, processing, and sampling. We performed all analyses by using 2-sided tests in Stata version 12.0 (StataCorp, https://www.stata.com) and considered p<0.05 statistically significant.

**Table 1:** Characteristics of persons >65 who received influenza vaccine versus those who did not receive influenza vaccine, Taiwan*

Characteristics	Before propensity score-matching				Propensity score-matched		
	Vaccinated	Unvaccinated	Standardized difference		Vaccinated	Unvaccinated	Standardized difference
No. patients	3,417,212	6,010,180			3,165,272	3,165,272	
Mean age, y (SD)	74.3 (6.4)	73.6 (6.8)	0.100		74.2 (6.4)	74.2 (6.9)	–0.002
Sex							
M	1,707,483 (50.0)	2,908,606 (48.4)	0.031		1,559,852 (49.3)	1,550,848 (49.0)	0.006
F	1,709,729 (50.0)	3,101,574 (51.6)			1,605,420 (50.7)	1,614,424 (51.0)	
Monthly income, $ Taiwan							
Dependent†	1,234,769 (36.1)	2,527,696 (42.1)	–0.122		1,190,930 (37.6)	1,197,102 (37.8)	–0.004
<19,100	794,513 (23.3)	1,365,600 (22.7)	0.013		728,908 (23.0)	721,281 (22.8)	0.006
19,100–41,999	1,367,022 (40.0)	2,054,058 (34.2)	0.121		1,224,632 (38.7)	1,226,438 (38.7)	–0.001
>42,000	20,908 (0.6)	62,826 (1.0)	–0.048		20,802 (0.7)	20,451 (0.6)	0.001
Urbanization‡							
Level 1	988,136 (28.9)	1,833,258 (30.5)	–0.035		926,242 (29.3)	925,049 (29.2)	0.001
Level 2	2,194,465 (64.2)	3,793,218 (63.1)	0.023		2,025,556 (64.0)	2,025,109 (64.0)	0.000
Level 3	199,301 (5.8)	320,751 (5.3)	0.022		180,635 (5.7)	181,562 (5.7)	–0.001
Level 4	35,310 (1.0)	62,953 (1.0)	–0.001		32,839 (1.0)	33,552 (1.1)	–0.002
No. outpatient visits in the previous 12 mo							
0–10	504,028 (14.7)	1,831,616 (30.5)	–0.383		504,014 (15.9)	500,316 (15.8)	0.003
11–20	869,805 (25.5)	1,598,542 (26.6)	–0.026		851,438 (26.9)	854,335 (27.0)	–0.002
21–30	776,389 (22.7)	1,106,821 (18.4)	0.107		719,783 (22.7)	721,771 (22.8)	–0.001
31–40	522,170 (15.3)	651,142 (10.8)	0.132		462,035 (14.6)	462,408 (14.6)	0.000
>40	744,820 (21.8)	822,059 (13.7)	0.214		628,002 (19.8)	626,442 (19.8)	0.001
CCI score (SD)§	7.7 (2.8)	7.2 (2.8)	0.236		7.8 (2.8)	7.8 (2.9)	–0.006
Underlying conditions							
Cerebrovascular disease	1,151,954 (33.7)	1,703,465 (28.3)	0.116		1,047,165 (33.1)	1,052,107 (33.2)	–0.003
Diabetes	1,377,596 (40.3)	2,000,525 (33.3)	0.146		1,246,943 (39.4)	1,252,696 (39.6)	–0.004
Hypertension	2,568,836 (75.2)	3,931,874 (65.4)	0.215		2,344,103 (74.1)	2,351,157 (74.3)	–0.005
CAD	1,672,350 (48.9)	2,340,127 (38.9)	0.203		1,520,516 (48.0)	1,447,015 (45.7)	0.047
Myocardial infarction	158,688 (4.6)	228,094 (3.8)	0.042		143,777 (4.5)	144,573 (4.6)	–0.001
PVD	238,514 (7.0)	324,691 (5.4)	0.065		217,314 (6.9)	210,393 (6.6)	0.009
Heart failure	555,349 (16.3)	792,171 (13.2)	0.087		503,135 (15.9)	506,674 (16.0)	–0.003
Dyslipidemia	1,557,151 (45.6)	2,231,936 (37.1)	0.172		1,436,998 (45.4)	1,340,441 (42.3)	0.062
Chronic liver disease	1,067,001 (31.2)	1,460,809 (24.3)	0.155		950,911 (30.0)	951,575 (30.1)	0.000
CKD	664,324 (19.4)	917,408 (15.3)	0.110		596,302 (18.8)	597,911 (18.9)	–0.001
Peptic ulcer disease	1,904,442 (55.7)	2,764,223 (46.0)	0.196		1,720,590 (54.4)	1,723,618 (54.5)	–0.002
Dementia	249,766 (7.3)	370,758 (6.2)	0.045		228,856 (7.2)	231,226 (7.3)	–0.003
Valvular heart disease	424,657 (12.4)	596,580 (9.9)	0.079		383,055 (12.1)	384,592 (12.2)	–0.001
Drug abuse	48,591 (1.4)	74,326 (1.2)	0.016		45,499 (1.4)	45,816 (1.4)	–0.001
Atrial fibrillation	160,148 (4.7)	235,626 (3.9)	0.038		146,674 (4.6)	147,989 (4.7)	–0.002
Medications							
Antiplatelet agents	559,272 (16.4)	735,104 (12.2)	0.118		491,897 (15.5)	493,185 (15.6)	–0.001
Insulin	42,022 (1.2)	58,973 (1.0)	0.024		38,581 (1.2)	38,823 (1.2)	–0.001
Oral diabetic drugs	399,409 (11.7)	552,935 (9.2)	0.081		358,531 (11.3)	361,189 (11.4)	–0.003
Diuretics	301,500 (8.8)	420,868 (7.0)	0.067		270,816 (8.6)	272,637 (8.6)	–0.002
Calcium channel blockers	651,895 (19.1)	892,880 (14.9)	0.113		580,984 (18.4)	583,106 (18.4)	–0.002
Beta-blockers	413,542 (12.1)	583,937 (9.7)	0.077		371,599 (11.7)	372,374 (11.8)	–0.001
ACEI/ARB	508,701 (14.9)	719,943 (12.0)	0.085		459,520 (14.5)	461,934 (14.6)	–0.002
Statins	167,188 (4.9)	249,822 (4.2)	0.035		155,133 (4.9)	155,624 (4.9)	–0.001
Propensity score	0.42 (0.13)	0.33 (0.14)	0.662		0.406 (0.129)	0.406 (0.129)	0.000

*Values are no. (%) except as indicated. ACEI, angiotensin-converting enzyme inhibitor; ARB, angiotensin II receptor blocker; CAD, coronary artery disease; CCI, Charlson Comorbidity Index; CKD, chronic kidney disease; PVD, peripheral vascular disease. †Dependent persons are those without an income. ‡Urbanization levels in Taiwan are divided into 4 strata according to the Taiwan National Health Research Institute publications. Level 1 designates the most urbanized areas, and level 4 designates the least urbanized areas. §CCI score is used to determine overall systemic health. Increased CCI scores are indicative of stepwise increases in the cumulative mortality.

## Results

Our study included 3,417,212 persons who received influenza vaccination and 6,010,180 who were not vaccinated during 12 consecutive influenza seasons. We matched demographic characteristics and baseline underlying conditions before and after propensity score matching (Table 1). Before propensity score matching, the vaccinated group was older (74.3 years of age) than the unvaccinated group (73.6 years of age) and had higher Charlson Comorbidity Index scores (7.7 + 2.8) than the unvaccinated group (7.2 + 2.8). The vaccinated group had higher rates of diabetes mellitus (40.3% vs. 33.3%) and coronary artery disease (48.9% vs. 38.9%) than the unvaccinated group. In addition, the vaccinated group had higher proportions of use of antiplatelet agents (16.4%) than the unvaccinated group (12.2%), and more used oral medications for diabetes (11.7%) than those in the unvaccinated group (9.2%). A total of 167,188 (4.9%) persons in the vaccinated group and 249,822 (4.2%) persons in the unvaccinated group were statin users.

Incidence rates of hospitalization for pneumonia and influenza increased over time. In 2000, the incidence rate was 26.71/1,000 person-years. By 2012, the incidence rate was 41.08/1,000 person-years (Table 2). During 2000–2012, hospitalization for pneumonia and influenza occurred on an average of 23,595 events per year (14,272–33,428 events/year).

**Table 2:** Incidence of hospitalization for pneumonia and influenza in persons >65 years of age during 2000–2012, Taiwan

Year	No. events	Person-years	Incidence/1,000 person-years
2000	14,272	534,348	26.71
2001	17,342	596,594	29.07
2002	17,383	615,868	28.23
2003	19,298	610,215	31.62
2004	21,807	640,872	34.03
2005	19,909	662,198	30.07
2006	22,394	664,194	33.72
2007	24,148	699,617	34.52
2008	25,829	713,500	36.20
2009	28,226	730,645	38.63
2010	31,945	733,967	43.52
2011	33,428	746,025	44.81
2012	30,760	748,838	41.08

Compared with the unvaccinated group, the vaccinated group had lower risks of in-hospital death (adjusted hazard ratio [aHR] 0.69, 95% CI 0.68–0.69), in-hospital death from pneumonia (aHR 0.72, 95% CI 0.70–0.73), hospitalization for pneumonia and influenza (aHR 0.84, 95% CI 0.84–0.85), hospitalization for circulatory conditions (aHR 0.90, 95% CI 0.90–0.90), and hospitalization for critical illnesses (aHR 0.75, 95% CI 0.74–0.76) (Table 3). For subgroup analyses stratified by statin use, the effects of vaccination on in-hospital death (*P*_interaction_ = 0.478), in-hospital death from pneumonia (*P*_interaction_ = 0.493), hospitalization for pneumonia and influenza (*P*_interaction_ = 0.138), hospitalization for circulatory condition (*P*_interaction_ = 0.667), and hospitalization for critical illness (*P*_interaction_ = 0.375) were consistent among statin users and nonusers. We also analyzed these data by using the Cox regression model, adjusted for propensity score only, and noted similar results (Appendix Table 2).

**Table 3:** Comparison of statin users and nonusers >65 years of age for incidence and risk for hospitalization, pneumonia, circulatory conditions, critical illness, and death who are vaccinated and unvaccinated for influenza, Taiwan*

Characteristics	Vaccinated				Unvaccinated (referent)				Hazard ratio (95% CI)†	
	No.	Person-years	Incidence‡		No.	Person-years	Incidence‡		Crude	Adjusted
In-hospital death§	38,320	2,984,344	12.84		55,405	2,949,054	18.79		0.68 (0.67–0.69)	0.69 (0.68–0.69)
Statin user	1,478	147,164	10.04		22,097	146,668	14.30		0.70 (0.66–0.75)	0.71 (0.67–0.76)
Statin nonuser	36,842	2,837,180	12.99		53,308	2,802,386	19.02		0.68 (0.67–0.69)	0.68 (0.68–0.69)
In-hospital death from pneumonia¶	15,057	2,984,931	5.04		20,699	2,950,157	7.02		0.72 (0.70–0.73)	0.72 (0.70–0.73)
Statin user	503	147,202	3.42		665	146,723	4.53		0.75 (0.67–0.85)	0.75 (0.67–0.85)
Statin nonuser	14,554	2,837,730	5.13		20,034	2,803,435	7.15		0.72 (0.70–0.73)	0.72 (0.70–0.73)
Hospitalization for pneumonia or influenza#	103,395	2,946,802	35.09		121,776	2,907,115	41.89		0.84 (0.83–0.84)	0.84 (0.84–0.85)
Statin user	3,967	145,687	27.23		4,810	144,805	33.22		0.82 (0.79–0.86)	0.82 (0.79–0.86)
Statin nonuser	99,428	2,801,115	35.50		116,966	2,762,309	42.34		0.84 (0.83–0.85)	0.84 (0.84–0.85)
Hospitalization for circulatory condition**	394,245	2,801,412	140.73		430,954	2,750,954	156.66		0.90 (0.90–0.90)	0.90 (0.90–0.90)
Statin user	25,141	134,914	186.35		27,991	132,843	210.71		0.89 (0.87–0.90)	0.90 (0.88–0.91)
Statin nonuser	369,104	2,666,498	138.42		402,963	2,618,111	153.91		0.90 (0.90–0.90)	0.90 (0.89–0.90)
Hospitalization for critical illness††	62,018	2,968,927	20.89		82,602	2,929,804	28.19		0.74 (0.73–0.75)	0.75 (0.74–0.76)
Statin user	2,614	146,432	17.85		3,574	145,677	24.53		0.73 (0.69–0.77)	0.74 (0.71–0.78)
Statin nonuser	59,404	2,822,496	21.05		79,028	2,784,127	28.39		0.74 (0.73–0.75)	0.75 (0.74–0.76)

*Values after propensity score-matching between persons who received and did not receive influenza vaccination. †Calculated by Cox regression model with adjusted imbalance covariates listed in Table 1. In all cases, p<0.001. ‡Per 1,000 person-years. §Interaction p = 0.478. ¶Interaction p = 0.493. #Interaction p = 0.138. **Interaction p = 0.667. ††Interaction p = 0.375.

## Discussion

In this nationwide population-based study in Taiwan, we investigated the effects of statin therapy on the risks for in-hospital death and severe complications of influenza infections in 9,427,392 persons >65 years of age during influenza seasons from 2000–01 through 2011–12. We found that vaccinated groups had lower risks than unvaccinated groups for in-hospital death; in-hospital death from pneumonia; and hospitalization for pneumonia and influenza, circulatory conditions, and critical illness. In the subgroup analysis stratified by statin use, the observed outcome differences across stain users and nonusers were consistent with chance.

Statins might exert antiinflammatory effects by inhibiting the major histocompatibility complex class II pathway of antigen presentation (*24*), preventing accumulation and recruitment of monocytes (*25*), reducing cytokine production by immune cells (*26*,*27*), and impairing the activation of T cells (*9*,*28*). Previous studies that directly address statin use and vaccine effectiveness have had conflicting results. A randomized clinical study of 150 healthy persons failed to find any difference in antibody responses to hepatitis A vaccine among those receiving atorvastatin and those receiving a placebo (*29*); that study was limited because only statin therapy initiated on the day of randomization, not prior chronic statin therapy, was considered. In contrast, another study of 105,874 vaccinated persons (39,342 statin users and 66,532 nonusers) and 141,714 unvaccinated persons (52,685 statin users and 89,029 nonusers) revealed reduced influenza vaccine effectiveness against acute respiratory illness among statin users compared with nonusers during influenza seasons (*14*). However, that study was limited by healthy-user bias (*30*,*31*) and did not match characteristics in controls.

Other large-scale studies have revealed suboptimal influenza vaccine effectiveness in persons >65 years of age because of age-related decline in the immune response, multiple underlying conditions, and concomitant medication use with possible secondary interactions (*32*,*33*). To address the potential negative effects of concomitant statin therapy on vaccine effectiveness, a prior post hoc analysis showed that influenza antibody titers were much lower in those receiving chronic statin therapy compared with those not receiving statin therapy (*13*). However, the association between antibody titers and adverse clinical outcomes was not characterized further, raising concern about the actual clinical implications. Another population-based retrospective cohort study of 1,403,651 Medicare beneficiaries >65 years of age in the United States matched statin users to nonusers and found that statin use does not dramatically affect the risk for influenza-related visits and influenza-related hospitalizations in this population (*15*). However, the study did not determine whether chronic statin use had any implication for major adverse cardiovascular events or death. 

We found that, among persons >65 years of age, vaccinated statin users and nonusers had lower risks of in-hospital death and severe complications of influenza infections compared with unvaccinated groups. In further analyses, we found no statistically significant difference and interaction between statin use and hospitalization for pneumonia, influenza, or circulatory conditions. However, vaccine effectiveness against critical illness slightly increased in statin users compared with nonusers, suggesting that the context of benefits of statins for cardiovascular outcomes could play a role for critically ill patients (*34*).

The strengths of our study include the use of a large nationwide population-based dataset, encompassing data from 9,427,392 patients >65 years of age. Our study covered 12 influenza seasons, from 2000–01 through 2011–12, aiding comparison of the effects of statins on the risks for death and hospital admission for major pulmonary and circulatory events in older vaccinated and unvaccinated persons. 

Our study has some limitations. First, relevant details enabling characterization of the geographic spread of influenza activity as sporadic, local, regional, or widespread, and information on influenza virus subtypes were not available in the NHIRD dataset. Therefore, we could not identify the effects of statin therapy on the spread of influenza. Second, we used data on persons registered in the national health insurance program, which included information on adverse clinical outcomes caused by local and widespread influenza, but we cannot rule out the possibility of diagnostic misclassification. Third, with a such large sample in our study, a statistical test would easily demonstrate a significant difference. However, the risk difference in the vaccinated group compared with the unvaccinated group ranged from 10% to 31%, and the difference is not small. For subgroup analyses, the overall treatment effects also are consistent across subsets of patients. In addition, although we explored the interaction between statin use and the effectiveness of the influenza vaccine, whether other drugs exerting similar pleiotropic effects, such as aspirin and nonsteroidal antiinflammatory drugs, or other vaccines commonly used in this population, including pneumococcal and herpes vaccines, have similar interactions is unknown. Finally, our study included only persons >65 years of age, and our results cannot be extrapolated to younger persons who often elicit stronger immune responses after vaccination.

In conclusion, influenza vaccination was associated with lower risks of in-hospital death and hospitalization for pulmonary and circulatory adverse outcomes in persons >65 years of age in Taiwan. Of note, the rate of hospitalization for critical illness was slightly lower in statin users than that for nonusers. These findings indicate that influenza vaccination should continue to be encouraged in older populations because it reduces disease-specific hospitalization and death. In addition, statin use might enhance the protective effects of the vaccine against critical illness.

Appendix Additional information on statin use and effectiveness of influenza vaccines in persons >65 years of age, Taiwan.
