# Supplementary material for: Statin Use and Influenza Vaccine Effectiveness in Persons >65 Years of Age, Taiwan
Source: Emerg Infect Dis. 2020 Jun;26(6):1243–50. doi: 10.3201/eid2606.190646 (PMC7258485; doi:10.3201/eid2606.190646)
Supplement: Appendix — Additional information on statin use and effectiveness of influenza vaccines in persons >65 years of age, Taiwan. [file 19-0646-Techapp-s1.pdf]

Article DOI: https://doi.org/10.3201/eid2606.190646

Statin Use and Influenza Vaccine Effectiveness in Persons >65 Years of Age, Taiwan

Appendix

Appendix Table. Propensity score model results of probability of influenza vaccination, Taiwan

Parameter	Estimate	Odds Ratios	95% CI	p value
Year of index date				
2000	Referent			
2001	0.9705	2.639	2.619–2.66	<0.0001
2002	1.4631	4.319	4.283–4.356	<0.0001
2003	–0.0701	0.932	0.924–0.941	<0.0001
2004	1.3956	4.037	4.004–4.071	<0.0001
2005	1.3768	3.962	3.93–3.995	<0.0001
2006	1.0452	2.844	2.821–2.867	<0.0001
2007	0.9147	2.496	2.475–2.517	<0.0001
2008	1.0588	2.883	2.859–2.907	<0.0001
2009	0.4734	1.606	1.592–1.619	<0.0001
2010	0.4704	1.601	1.587–1.614	<0.0001
2011	0.6513	1.918	1.902–1.934	<0.0001
2012	0.7056	2.025	2.008–2.042	<0.0001
Age, per year	0.0105	1.011	1.01–1.011	<0.0001
Sex				
M	0.1111	1.118	1.114–1.121	<0.0001
F	Referent			
Monthly income, $ Taiwan				
Dependent†	Referent			
<19,100	0.0998	1.105	1.101–1.109	<0.0001
19,100–42,000	0.3011	1.351	1.347–1.356	<0.0001
>42,000	–0.3458	0.708	0.697–0.719	<0.0001
Urbanization‡				
Level 1	Referent			
Level 2	0.0376	1.038	1.035–1.041	<0.0001
Level 3	0.0458	1.047	1.04–1.053	<0.0001
Level 4 (rural)	–0.0108	0.989	0.976–1.002	0.1061
Number of outpatient visits in 12 mo.				
0–10	Referent			
11–20	0.6247	1.868	1.86–1.876	<0.0001
21–30	0.8388	2.314	2.303–2.324	<0.0001
31–40	0.9532	2.594	2.581–2.607	<0.0001
>40	1.0869	2.965	2.951–2.979	<0.0001
Charlson Comorbidity Index score§	–0.018	0.982	0.981–0.983	<0.0001

Parameter	Estimate	Odds Ratios	95% CI	p value
Underlying conditions				
Cerebrovascular disease	–0.00079	0.999	0.996–1.003	0.6491
Diabetes	0.1092	1.115	1.111–1.119	<0.0001
Hypertension	0.1818	1.199	1.195–1.203	<0.0001
Coronary artery disease				
Myocardial infarction	–0.0193	0.981	0.974–0.987	<0.0001
Heart failure	–0.0392	0.962	0.958–0.966	<0.0001
Chronic liver disease	0.1682	1.183	1.179–1.187	<0.0001
Chronic kidney disease	0.0454	1.046	1.042–1.051	<0.0001
Peptic ulcer disease	0.1579	1.171	1.167–1.175	<0.0001
Valvular heart disease	0.0614	1.063	1.059–1.068	<0.0001
Drug abuse	0.0215	1.022	1.01–1.034	0.0003
Atrial fibrillation	–0.0717	0.931	0.925–0.937	<0.0001
Medication				
Anti-platelet agents	0.0885	1.092	1.088–1.097	<0.0001
Insulin	–0.0815	0.922	0.911–0.933	<0.0001
Oral diabetic drugs	–0.0156	0.984	0.98–0.989	<0.0001
Diuretics	–0.077	0.926	0.921–0.931	<0.0001
Calcium channel blockers	0.022	1.022	1.018–1.026	<0.0001
Beta-blockers	–0.00866	0.991	0.987–0.996	0.0001
ACEI/ARB	–0.00128	0.999	0.994–1.003	0.5643
Statins	0.0469	1.048	1.041–1.055	<0.0001

*ACE, angiotensin-converting enzyme; ARB, angiotensin II receptor blocker.

†Dependent persons are those without an income.

‡Urbanization levels in Taiwan are divided into 4 strata according to the Taiwan National Health Research Institute publications. Level 1 designates the most urbanized areas, and level 4 designates the least urbanized areas.

§Charlson Comorbidity Index (CCI) score is used to determine overall systemic health. Increased CCI scores are indicative of stepwise increases in the cumulative mortality.

Appendix Table 2. Comparison of statin users and nonusers >65 years of age for incidence and risk for hospitalization, pneumonia, circulatory conditions, critical illness, and death who are vaccinated and unvaccinated for influenza, Taiwan

	Vaccinated			Unvaccinated (referent)			Hazard ratio (95% CI)*	
Characteristics	No.	Person-years	Incidence†	No.	Person-years	Incidence†	Crude	Adjusted
In-hospital death‡	41,566	3,222,938	12.90	88,003	5,580,167	15.77	0.62 (0.81–0.83)	0.69 (0.68–0.70)
Statin user	1,577	158,645	9.94	3,077	235,388	13.07	0.76 (0.72–0.81)	0.71 (0.67–0.75)
Statin nonuser	39,989	3,064,292	13.05	84,926	5,344,779	15.89	0.82 (0.81–0.83)	0.69 (0.68–0.70)
In-hospital death from pneumonia§	16,316	3,223,563	5.06	32,601	5,582,287	5.84	0.87 (0.85–0.88)	0.72 (0.71–0.74)
Statin user	531	158,686	3.35	967	235,462	4.11	0.81 (0.73–0.91)	0.74 (0.67–0.83)
Statin nonuser	15,785	3,064,877	5.15	31,634	5,346,825	5.92	0.87 (0.85–0.89)	0.72 (0.71–0.74)
Hospitalization for pneumonia and influenza¶	114,437	3,181,268	35.97	192,304	5,515,613	34.87	1.03 (1.03–1.04)	0.85 (0.84–0.85)
Statin user	4,335	157,020	27.61	6,997	232,714	30.07	0.92 (0.88–0.96)	0.83 (0.80–0.86)
Statin nonuser	110,102	3,024,248	36.41	185,307	5,282,899	35.08	1.04 (1.03–1.05)	0.85 (0.84–0.85)
Hospitalization for circulatory condition#	439,030	3,018,404	145.45	682,798	5,271,935	129.52	1.12 (1.12–1.13)	0.90 (0.90–0.91)
Statin user	27,573	145,156	189.95	41,536	215,031	193.16	0.98 (0.97–1.00)	0.90 (0.88–0.91)
Statin nonuser	411,457	2,873,248	143.20	641,262	5,056,903	126.81	1.13 (1.13–1.14)	0.90 (0.90–0.91)
Hospitalization for critical illness**	68,861	3,205,876	21.48	130,431	5,550,615	23.50	0.91 (0.91–0.92)	0.76 (0.75–0.76)
Statin user	2,832	157,855	17.94	5,271	233,910	22.53	0.80 (0.76–0.83)	0.74 (0.70–0.77)
Statin nonuser	66,029	3,048,021	21.66	125,160	5,316,705	23.54	0.92 (0.91–0.93)	0.76 (0.75–0.77)

*Adjusted for propensity score. p<0.001 in all instances.

†Per 1,000 person-years.

‡Interaction p = 0.747.

§Interaction p = 0.701.

¶Interaction p = 0.073.

#Interaction p = 0.492.

**Interaction p = 0.024.
